# Correction to: Hypotonicity-induced cell swelling activates TRPA1

**DOI:** 10.1007/s12576-019-00662-z

**Published:** 2019-02-08

**Authors:** Fumitaka Fujita, Kunitoshi Uchida, Yasunori Takayama, Yoshiro Suzuki, Masayuki Takaishi, Makoto Tominaga

**Affiliations:** 1 Division of Cell Signaling, Okazaki Institute for Integrative Bioscience (National Institute for Physiological Sciences), National Institutes of Natural Sciences, Okazaki, Aichi 444-8787 Japan; 2 Basic Research Institute, Mandom Corp., Osaka, 540-8530 Japan; 3 Department of Physiological Sciences, SOKENDAI (The Graduate University for Advanced Studies), Okazaki, Aichi 444-8585 Japan; 4 Laboratory of Advanced Cosmetic Science, Graduate School of Pharmaceutical Sciences, Osaka University, 1-6 Yamadaoka, Suita, Osaka, 565-0871 Japan; 5 Product Assurance Division, Mandom Corp., Osaka, 540-8530 Japan; 6 Institute for Environmental and Gender-Specific Medicine, Juntendo University, Tokyo, 113-0033 Japan

## Correction to: J Physiol Sci (2018) 68:431–440 10.1007/s12576-017-0545-9

The article Hypotonicity-induced cell swelling activates TRPA1, written by Fumitaka Fujita, Kunitoshi Uchida, Yasunori Takayama, Yoshiro Suzuki, Masayuki Takaishi and Makoto Tominaga, was originally published electronically on the publisher’s internet portal (currently SpringerLink) on 16 June 2017 without open access.

With the author(s)’ decision to opt for Open Choice the copyright of the article changed on 28 January 2019 to © The Author(s) [Year] and the article is forthwith distributed under the terms of the Creative Commons Attribution 4.0 International License (http://creativecommons.org/licenses/by/4.0/), which permits use, duplication, adaptation, distribution and reproduction in any medium or format, as long as you give appropriate credit to the original author(s) and the source, provide a link to the Creative Commons license and indicate if changes were made.

